# Detecting tail biters by monitoring pig screams in weaning pigs

**DOI:** 10.1038/s41598-024-55336-7

**Published:** 2024-02-24

**Authors:** Philipp Heseker, Tjard Bergmann, Marina Scheumann, Imke Traulsen, Nicole Kemper, Jeanette Probst

**Affiliations:** 1 Institute for Animal Hygiene, Animal Welfare and Farm Animal Behavior (ITTN), University of Veterinary Medicine Hannover, Foundation, Hannover, Germany; 2 Department of Animal Sciences, Livestock Systems, Georg-August-University Goettingen, Göttingen, Germany; 3 Institute for Zoology, University of Veterinary Medicine Hannover, Foundation, Hannover, Germany; 4 Institute of Animal Breeding and Husbandry, Christian-Albrechts-University Kiel, Kiel, Germany

**Keywords:** Animal behaviour, Behavioural ecology

## Abstract

Early identification of tail biting and intervention are necessary to reduce tail lesions and their impact on animal health and welfare. Removal of biters has become an effective intervention strategy, but finding them can be difficult and time-consuming. The aim of this study was to investigate whether tail biting and, in particular, individual biters could be identified by detecting pig screams in audio recordings. The study included 288 undocked weaner pigs housed in six pens in two batches. Once a tail biter (n = 7) was identified by visual inspection in the stable and removed by the farm staff, the previous days of video and audio recordings were analyzed for pig screams (sudden increase in loudness with frequencies above 1 kHz) and tail biting events until no biting before the removal was observed anymore. In total, 2893 screams were detected in four pens where tail biting occurred. Of these screams, 52.9% were caused by tail biting in the observed pen, 25.6% originated from other pens, 8.8% were not assignable, and 12.7% occurred due to other reasons. In case of a tail biting event, screams were assigned individually to biter and victim pigs. Based on the audio analysis, biters were identified between one and nine days prior to their removal from the pen after visual inspection. Screams were detected earlier than the increase in hanging tails and could therefore be favored as an early warning indicator. Analyzing animal vocalization has potential for monitoring and early detection of tail biting events. In combination with individual marks and automatic analysis algorithms, biters could be identified and tail biting efficiently reduced. In this way, biters can be removed earlier to increase animal health and welfare.

## Introduction

Tail biting is a multifactorial problem in intensive pig farming and reduces animal health and animal welfare with huge economic losses. Many different strategies are implemented to prevent this behavioral disorder^1^. Docking the pigs’ tail in the first week of life has become a common method to prevent tail biting, but is discussed controversially due to the pain- and stressful procedure itself and potential effects such as chronic pain occurring later^2^. Although tail biting occurs in pigs with intact and with docked tails, tail docking is very effective for reducing tail lesions^2–5^. In general, in accordance with the European Union (EU) Council Directive 120/2008/EC, routine tail docking is prohibited in the EU and only allowed if other preventive measures have failed^6^. However, with an official certificate of exemption by the authorities it was performed on average in 77% (median = 95%) of the pig population in the EU^6^. Risk factors influencing the occurrence of tail biting are, amongst others, a high stocking density, genetics, restricted feeder space, and deficiencies in feed quality, indoor climate, disease outbreaks, and the lack of adequate enrichment material in the barren environment^6–11^. To enable a timely intervention of tail biting events early detection is crucial but challenging. Early indicators are increased activity^12–15^, changes in the tail posture from curly to hanging or stuck tails^12,16–21^, different drinking or feeding behavior^22,23^, and an increased object manipulation^13,24^.

Three different tail biting types are described in pig husbandry^25^. The two-stage tail biting where pigs gently explore or chew other pigs’ tails (pre-damage stage) with no visible damage, usually due to a lack of enrichment material to root, chew, or manipulate, before the skin breaks and harmful biting (damaging stage) with serious tail damage starts^26^. The sudden-forceful tail-biting that occurs as a reaction to the absence of or limited access to resources like water and feed^25,26^ and the obsessive tail biting where specific animals in a group focus on forcefully biting other pigs’ tails^25,27^. The causes for this latter type of behavior remain unclear, but are possibly genetically determined with an individual problem of the protein metabolism, which results in a higher attraction to blood^28^. Once tail biting starts among pigs, detecting and removing biters is an important intervention strategy^11,15,25,27^. However, due to changed animal behavior when humans enter the compartment, identification can be difficult and time-consuming.

The implementation of Precision Livestock Farming (PLF) tools is gaining more and more attention because of the objective and continuous monitoring of animals^29–31^. Especially the use of image analysis for automatic monitoring of pigs’ activity^32–36^ or tail posture^37,38^ by using machine vision and deep learning algorithms seem to be promising options for early identification of indicators for tail biting and for developing early warning tools.

Analyzing animal vocalization has become a promising approach for objective monitoring of animal health and welfare in different situations^39^. The vocalization can be used for determining positive and negative emotions^40,41^, detection and localization of coughing^42,43^, or identifying piglets being crushed^44^. Moreover, high frequency pig screams can be used for detecting and monitoring the occurrence of ear biting^45^ and can be automatically detected from audio data and used as a reliable tool to distinguish stressful situations by training neural networks^46,47^. Especially screams during nighttime indicate serious stressful situations, as pigs are usually resting in that interval. In contrast, during daytime more stress calls are expected due to them competing for access to feed^47^. However, to our knowledge, no study has used pig screams as a tool for detecting tail biting and for identifying biting pigs for early separation so far.

Therefore, the aim of this study was to investigate whether the detection of pig screams can be used for identifying tail biting events in weaner pigs under practical conditions to enable faster intervention strategies such as removing the biter from the pen.

## Methods

### Ethics declarations

The study was conducted at the experimental station for pig farming of the Chamber of Agriculture Lower Saxony in Bad Zwischenahn-Wehnen in north-west Germany from March 2022 until July 2022. All experiments were performed and all animals were housed strictly in accordance with European guidelines (EU Council Directive 2008/120/EC), German legislation (German Animal Welfare Act and German Order for the Protection of Production Animals Used for Farming Purposes and Other Animals Kept for the Production of Animal Products) and complied with the ARRIVE guidelines (Animal Research: Reporting of In Vivo Experiments). No invasive procedures in the animals were carried out. The trials were reviewed and approved by the Ethics Committee of the Chamber of Agriculture Lower Saxony (approval number: A21-TS21923-LWK-3031-1).

### Animals and husbandry

One compartment with six pens for weaner pigs (24 pigs/pen) was used for investigations in two batches. Animal husbandry was in accordance with the EU and German legislation. In total, 288 weaner pigs were included in the experiment. Piglets (Pietrain x [Large White x Landrace]) were born under conventional housing conditions on the farm, males were castrated within the first week of life, and tails were kept undocked. At weaning (26.48 ± 1.67 days of age), pigs were weighed individually (7.28 ± 1.35 kg) and received a colored ear tag (PRIMAFLEX size 0 or 1, Caisley, Bocholt, Germany) for individual identification before being moved to the rearing compartment where they remained for 39 days. Only pigs weighing more than 5 kg and having a full length tail were included in the experiment. Piglets of the same litter were kept together as far as possible to avoid fighting and minimize weaning stress. A maximum of three litters were mixed to fill one pen in the rearing compartment. Each rearing pen measured 2.65 × 5.00 m and had a fully slatted floor that was divided into one third iron (dunging area) and two thirds plastic slatted flooring. Two drinking stations with two nipple drinkers, a dry feeder (2.4 m wide), and sisal ropes as basic enrichment material were offered in every pen. Additionally, an automatic controlled shower system (Sprock Agrarsysteme, Bösel, Germany) and an automatic enrichment device (IBO Stalltechnik GmbH, Rhede, Germany) were installed in each pen for regularly moistening the dunging area and providing organic enrichment material (alfalfa pellets or oat bran pellets) to the pigs for exploration and manipulation. Room temperature in the forced-ventilated compartment was set at 28 °C at the start of the rearing period and evenly reduced to 23 °C at the end. For the first six days, pigs were fed ad libitum with a creep feed (RicoWean, AGRAVIS Raiffeisen AG, Oldenburg, Germany). For the following two weeks the feed was changed to a commercial rearing diet (EuroStart, AGRAVIS Raiffeisen AG). Then, until the animals were transferred to the fattening compartment, they were fed with another commercial diet (VincoStart, AGRAVIS Raiffeisen AG). Diet transition was done gradually over five days for better acceptance and physiologic adaptation. Natural lighting was provided by windows, and artificial lighting was switched on from 07:30 until 17:30.

### Data recording

Each pen was equipped with a video camera (AXIS M3206-LVE Network Camera, Axis Communications AB, Lund, Sweden) connected to an audio module (AXIS T6101 Audio and I/O Interface, Axis Communications AB) and a microphone (AXIS T8351 Mk II, Axis Communications AB). Then, the cameras were connected to a NAS-Server (Synology DS720+, New Taipei City, Taiwan) for storing videos. They were mounted approximately 2.8 m above the floor with top-down view of the entire pen. There, they recorded continuously one-hour long video files with a resolution of 1920 × 1080 pixels and a frame rate of 20 frames per second (FPS) during the rearing period. Microphones (frequency range 20 Hz – 20 kHz) were hung from the ceiling in the middle of the pen at a height of approximately 1.2 m above the floor and recorded audio with a sampling frequency of 16 kHz.

Individual tail examinations were carried out biweekly using the German Pig Scoring Key (DSBS)^48^ by two trained observers who entered the pen to score every tail. Tail lesions were recorded by using a four-point scoring system (0 = no lesion, 1 = superficial lesion, 2 = small lesion, 3 = large lesion). For further evaluation, the sum of tail lesion scores for each pen was used. Moreover, tail posture (number of hanging tails in the pen) and fresh tail lesions were recorded daily from Monday through Friday at pen level. In case of a tail biting outbreak (at least one bleeding tail in the pen), extra enrichment material (jute bags, straw, bite stars) was provided for enabling additional exploration to reduce the tail biting and therefore to increase animal welfare. When tail lesions in the pen increased, the farm staff observed the pen during their daily inspections and as soon as a pig showed special interest in biting and was seen at least two times aggressively biting the tail of other pigs (victim), this pig was defined as a biter and removed from the pen by the farm staff to maintain animal welfare. During the two batches, seven pigs in four pens were identified by above criteria as tail biters (1–2 biters/pen) and separated from the group.

### Data analyzing

**Figure 1:**
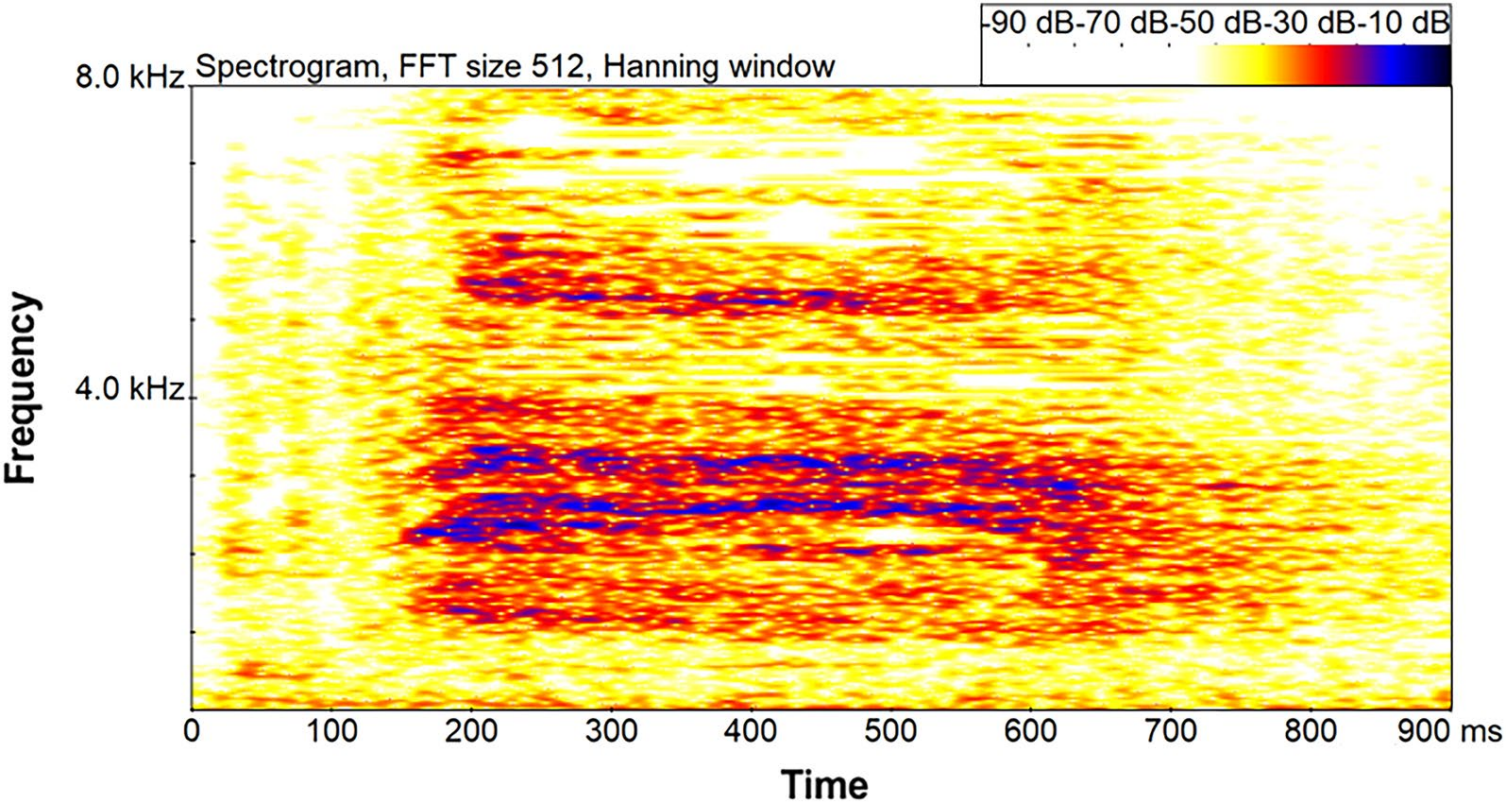
Pig scream due to a tail biting event shown in the spectrogram of the audio data. The figure is created in BatSound^®^ Version 4.2.1 (URL: https://batsound.com/product/batsound/).

After identification and removal of the biter from the pen by the farm staff, videos were analyzed retrospectively and continuously by one observer. Audio files were extracted from the video recordings and analyzed for pig screams (Audacity^®^, Version 3.1.3, Audacity Team). Potential pig screams were characterized by a sudden increase in loudness amplitude with high energy levels in frequencies above 1 kHz and were clearly visible in the oscillogram and spectrogram of the audio file (Fig. 1). The increase varied due to the variable distance between the screaming pig and the microphone. The observer listened to each potential scream to ensure that it was not a different sound. The duration of the screams was measured as the time from onset to offset of a call. The time stamp of the scream in the audio file was documented (Microsoft Excel 2016, Microsoft Corporation, One Microsoft Way, Redmond, WA, USA), and then it was evaluated in the video file to determine the reason for the scream and the exact time of day it occurred (VLC media player 3.0.16 Vetinari, VideoLAN, Paris, France). Screams were labeled and assigned to different categories in an ethogram (Table 1).

**Table 1:** Ethogram of categories for assigning causes to detected pig screams to determine their origins during the labeling process.

Category	Description
Foot on tail	A pig steps with its foot on a lying or sitting pig’s tail
Human contact	Farm staff is in the pen to examine or treat pigs
Mounting	A pig jumps with its front legs on another pig’s back
Other pen	Scream clearly originates from a different pen
Scream-detected tail biting	A pig chews and forcefully bites into another pig’s tail, resulting in a response reaction of the bitten pig with a scream
Other pig manipulation	A pig manipulates any part of another pig’s body apart from the tail (e.g., belly nosing), or two pigs fight in a pen
Pig being frightened	Pigs in a pen are frightened for an unknown reason and start running through the pen
Competition over feed	Pigs fight and compete to access feed
Not assignable	Scream was not assignable
Removal of biter	A biter is removed from the pen by the farm staff

In case of a scream-detected tail biting event during daytime, the biting (biter) and the bitten (victim) pig were identified by the individual colored ear tag in the video. During the nighttime, no individual pig identification was possible due to the cameras’ infrared night mode. Thus, nighttime biting events were only used to label different context categories. Retrospectively, we inspected audio and video files of the days prior to removing the biters by visual inspection. The files were continuously analyzed until no tail biting by the removed biters was observed anymore for one day (24 h). The day on which the last biter was removed from a pen was defined as day 0 (d0), days before the removal were indicated with negative numbers (e.g., d-1). Days after the removal of a biter got positive numbers (e.g., d1). A total of 748 h of video recording material were analyzed and categorized for this study.

### Statistical analysis

Screams were documented in Microsoft Excel 2016 (Microsoft Corporation, One Microsoft Way) for further descriptive analysis of affiliation to the different categories and their duration. The number of scream-detected tail bites per hour and day was calculated by adding up the number of detected screams caused by tail biting. Hours per day were summarized (Daytime), including all hours from 07:00 through 18:00, and hours per night (Nighttime). Total screams (including daytime and nighttime calls) and screams caused by the removed biters (biters 1–7) in the different pens before their removal were investigated. Screams caused by pigs other than the removed biters during the day were summarized as “other pigs”. Scream-detected tail bites per hour from the different biters were calculated, but only hours with at least one recorded tail bite were evaluated. The duration of screams was compared between the different categories for the scream’s origin. Data (scream-detected tail bites per hour and duration of screams) were tested for normal distribution by the Shapiro–Wilk test and analyzed with the Kruskal–Wallis and the post-hoc Dunn’s all-pairs test for significant differences due to the missing normal distribution.

To investigate whether time before removal and daytime affected the scream-detected tail biting activity, a binomial generalized linear mixed-effects model (GLMM) for the occurrence of tail biting per hour and a linear mixed effects model for the number of bites per hour were calculated. Thereby, the occurrence of scream-detected tail biting for each hour (present = at least one tail biting event, or not present), or the number of tail bites per hour were used as response variables and Day (= day before removal) and Daytime (Day, Night) as well as an interaction of both as test variables while controlling for pen as random effect. For the linear mixed-effects (LME) model, the number of biters (1, 2) was additionally included as a test variable. To achieve normal distribution, the number of scream-detected tail bites was transformed by using the fourth root^49^. Afterwards, it was checked whether the assumption of normal distribution for the LME model were fulfilled by checking the residuals of the model using histograms and the Q-Q plot. For both models, stepwise backward elimination was used to establish the best fitting model based on the full model. The highest-level interaction term with the highest non-significant p-value was removed from the previous model and afterwards, at each step the models were compared using Wald test statistics (anova function) until a significant difference between the reduced and the previous model was indicated, or only main terms remained in the model. The significance level was set to p < 0.05 in every test. All tests were performed in R (Version 4.3.0)^50^ and RStudio (Version 2023.06.0 Build 421)^51^, including the PMCMRplus package^52^, the lme4 package^53^, the nlme package^54^ and the ggplot2 package^55^ for visualizations. Tail lesion scores were added together at pen level as the sum of tail lesion scores (0–3) for the biweekly scoring days. The tail posture was shown as the percentage of pigs in a pen with their tail hanging down or tucked between their legs.

## Results

### Individual differences in tail biting activity

During daily visual inspections, the farm staff identified seven intensive biters from four pens in the two batches with weaner pigs and separated them from the group. Interestingly, those biters were not the first pigs that started tail biting in the analyzed videos, but started biting on the following day. In pen 1, a total of seven biting pigs were detected, compared to 15 pigs in pen 2, 10 pigs in pen 3, and 15 pigs in pen 4. A total of 2893 pig screams were detected in the video recordings of the four tail biting pens and videos were analyzed up to 10 days prior the removal of a biter (Table 2).

**Table 2:** Tail biting activity detected by audio analysis of the seven removed biters in the different pens during the observation periods.

	Biter						
	1	2	3	4	5	6	7
Pen	1	1	2	2	3	4	4
Day of first biting event before removal	3	4	1	8	4	9	4
Total scream-detected biting events	97	45	19	143	244	279	42
Max. scream-detected biting events per day	30	22	18	129	107	58	23
Max. scream-detected biting events per hour	12	9	13	33	25	20	8

**Figure 2:**
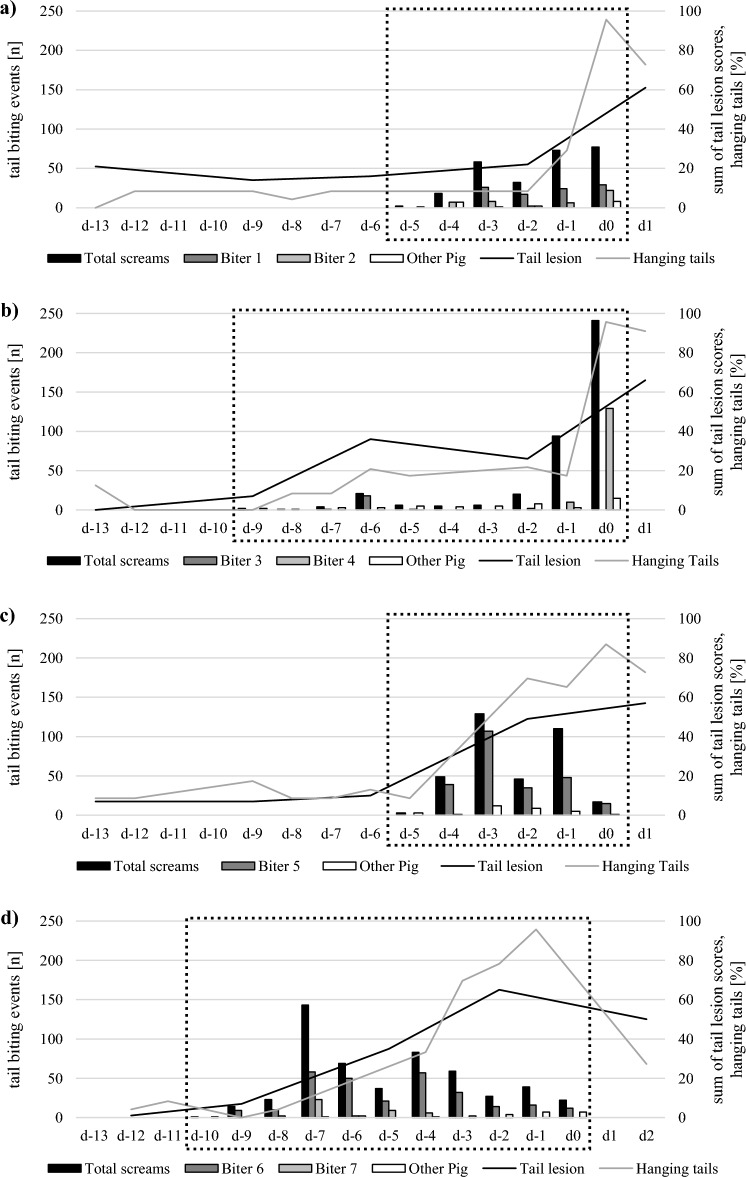
Detected pig screams caused by tail biting events (n = 1531) and biters (1–7) for the specified days (d) of the observation period (indicated by dotted lines), and sum of tail lesion scores and percentage of hanging tails in the different pens; (**a**) pen 1, (**b**) pen 2, (**c**) pen 3, (**d**) pen 4. The figure is created in Microsoft Excel 2016 (Microsoft Corporation, One Microsoft Way).

Tail biting started slowly with one to three performed scream-detected tail biting events on the first day of observation in the four pens (Fig. 2). Peaks and development of scream-detected biting events varied between the pens (Supplementary Figure S1).

**Figure 3:**
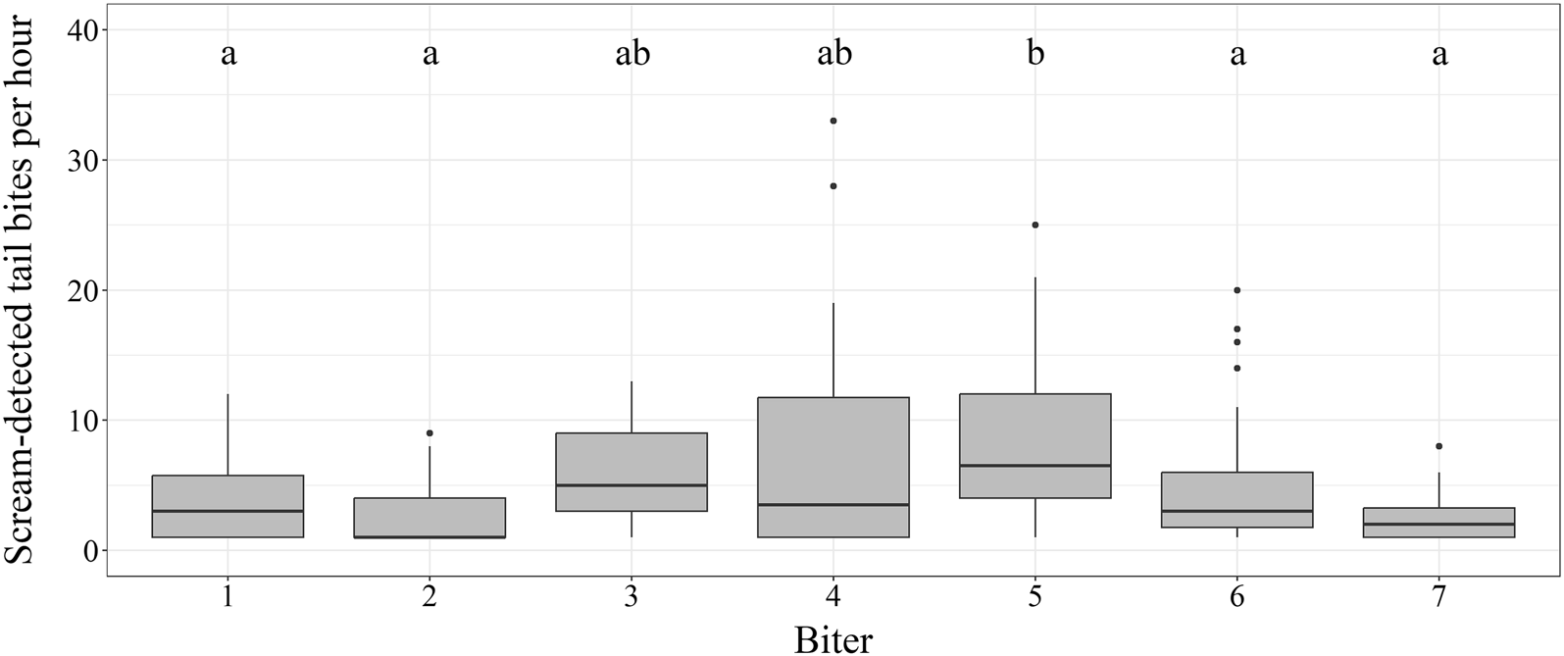
Scream-detected tail bites per hour during the observation period for the hours where at least one tail biting event occurred for biter 1 (n = 26), biter 2 (n = 17), biter 3 (n = 3), biter 4 (n = 18), biter 5 (n = 30), biter 6 (n = 64), and biter 7 (n = 16). Significant differences (p < 0.05) were found between the biters and indicated by different letters (**a,b**). The figure is created in R (Version 4.3.0)^50^ (https://www.r-project.org/) and RStudio (Version 2023.06.0 Build 421)^51^ (https://posit.co/download/rstudio-desktop/), using the ggplot2-package^55^ (https://ggplot2.tidyverse.org/).

Intensity of scream-detected tail biting differed significantly (p < 0.05) and showed individual variance between the seven biters during the observed hours with tail biting (n = 174). The highest total number of screams due to tail bites was recorded for biter 6 (pen 4) with 279 tail bites and biter 5 (pen 3) with 244 tail bites during the observation period. The lowest number of tail bites was performed by biter 3 (pen 2) with 19 detected events due to prompt identification and separation. The highest number of tail bites per hour was observed for biter 4 (pen 2) with 33 tail bites and biter 5 with 25 tail bites (Fig. 3).

**Figure 4:**
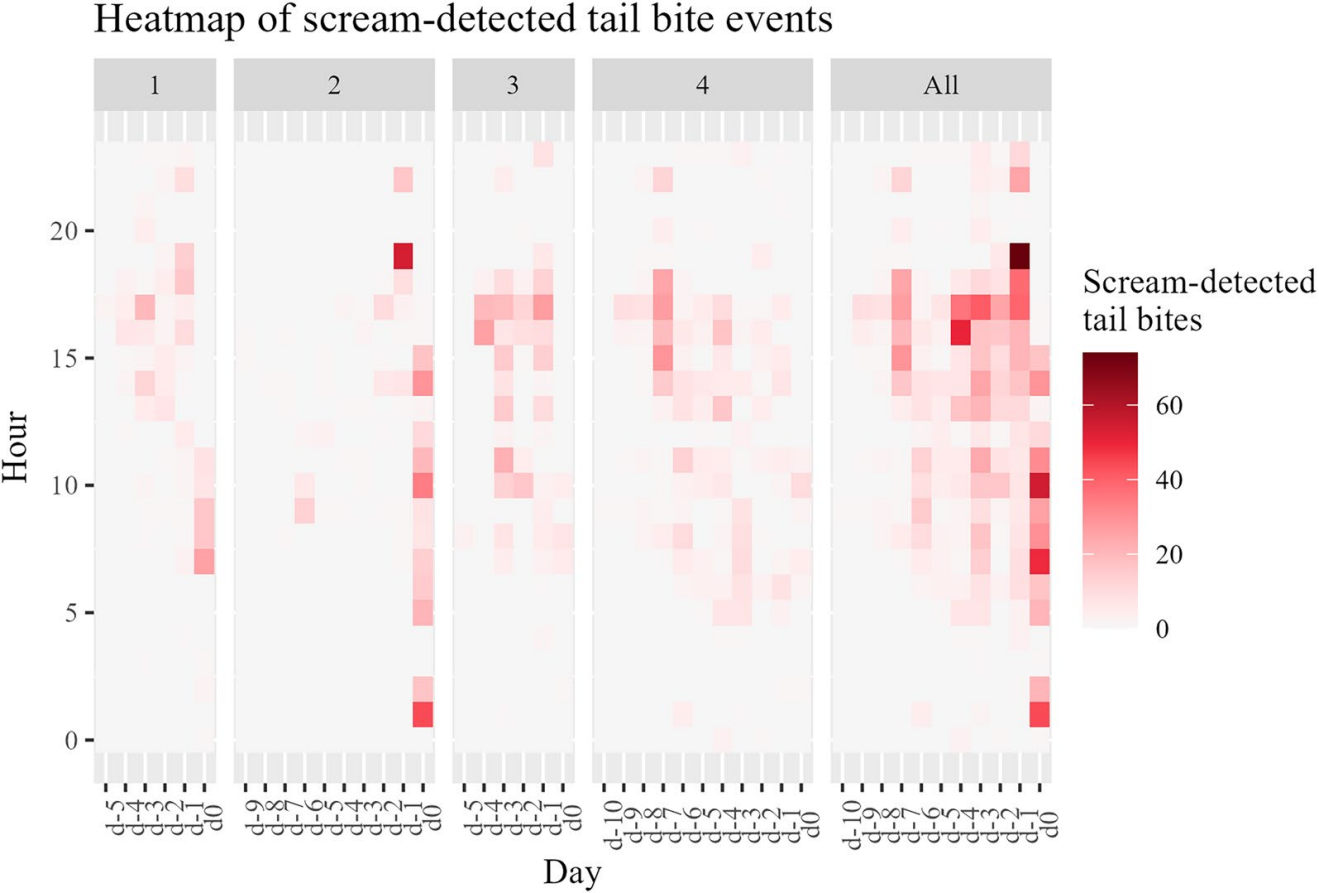
Heatmap of the observed tail biting activity (scream-detected tail bites per hour) due to the detected screams during the day in each of the four pens (1–4) and in total for all pens (all) during the analyzed days. The figure is created in R (Version 4.3.0)^50^ (https://www.r-project.org/) and RStudio (Version 2023.06.0 Build 421)^51^ (https://posit.co/download/rstudio-desktop/), using the ggplot2-package^55^ (https://ggplot2.tidyverse.org/).

Different patterns of scream-detected tail bites per hour during the observation period of the four pens were observed, showing hours with high tail biting activity due to the detected screams and hours with no screams on the observed days (Fig. 4).

### Context in which screaming occurs

Most screams (52.92%) originated from tail biting events, with 34.26% of the screams caused by tail biting during the daytime (with biter/victim identification) and 18.67% because of tail biting at nighttime (Table 3). About a quarter of the screams (25.61%) derived from another pen, but were clearly audible, followed by 8.78% of the screams that were not assignable to any one of the categories. Pigs stepping with their foot on another pig’s tail was the reason for 4.63% of the screams, and 3.15% occurred due to mounting behavior. Other pig manipulation (e.g., extreme belly nosing or fighting) was the reason for 2.07% of the screams, competing for feed for 1.14%, human contact for 1.07%, removal of biter for 0.38%, and frightened pigs for 0.24% of the detected screams.

**Table 3:** Number of screams in the observation period assigned to the different pens and categories of the ethogram.

	Pen			
	1	2	3	4
Number of analyzed days	5	9	5	10
Removal of biter	3	2	1	5
Competition over feed	1	5	0	27
Foot on tail	0	8	14	112
Human contact	1	12	9	9
Mounting	3	26	24	38
Not assignable	4	60	52	138
Other pen	103	267	144	227
Other pig manipulation	9	16	13	22
Pig being frightened	1	4	0	2
Scream-detected tail biting in the daytime	160	210	275	346
Scream-detected tail biting in the nighttime	100	190	79	171
Total number of screams	385	800	611	1097

### Scream duration

The mean scream ranged from 0.4 s up to 3.5 s. No significant differences (p > 0.05) between the two categories tail biting in the daytime and tail biting in the nighttime were found, but those durations differed significantly (p < 0.05) from the categories foot on tail, human contact, mounting, other pen, other pig manipulation, and not assignable (Table 4). The shortest duration (0.4 s) was seen when pigs were frightened by an unknown reason. Regarding the screams caused by tail biting, the duration ranged from 0.1 s to 4.2 s. The longest scream lasted 29.5 s and originated from another pen so no assignment was possible. A high variation was observed in all categories, which indicated the difficulty in using only the duration for assigning screams to different contexts.

**Table 4:** Mean scream duration of the different categories (p < 0.05).

Category	n	Average scream duration (seconds)*	Standard deviation (seconds)
Foot on tail	134	0.6^bde^	0.3
Human contact	31	1.1^de^	2.7
Mounting	91	0.7^bde^	1.0
Other pen	741	0.8^be^	1.7
Scream-detected tail biting in the daytime	991	0.8^ac^	0.6
Scream-detected tail biting in the nighttime	540	0.9^ac^	0.7
Other pig manipulation	60	0.6^bde^	1.0
Pig being frightened	7	0.4^bcde^	0.2
Competition over feed	33	1.3^abc^	1.7
Not assignable	254	0.6^d^	0.9
Removal of biter	11	3.5^a^	2.9

*Significant differences (p < 0.05) were found between the categories and indicated by different letters (a,b,c,d,e).

### Effect of daytime and time until removal

The final GLMM and LME models consisted of main effects only. Thus, the day (p < 0.0001) and daytime (p < 0.0001) significantly affected the occurrence of scream-detected tail biting and the number of tail bites per hour. Additionally, the number of biters had a significant influence (p = 0.0002) on the number of scream-detected tail bites per hour in the LME model. Significantly more scream-detected tail bites were observed in the daytime than in the nighttime (p < 0.0001) and in a pen with two biters compared to only one biter (p = 0.0002).

### Tail lesion scores and tail posture

Tail lesion scores and the percentage of hanging tails increased rapidly in all pens from the day of detecting the first biting event in the video until the removal of the biter (Fig. 2). After removing the biters, the sum of tail lesion scores decreased within a few days. No statement can be made regarding pen 2 due to the late removal of the second biter and the end of the rearing period when all pigs were moved to a fattening compartment.

The percentage of hanging tails reached a maximum on the day of separation. In pen 1, eight days after separation of the two biters, the remaining pigs adopted curly tail postures again with the number of hanging tails decreasing to 36.36% (d8).

## Discussion

This study aimed to explore the potential for using pig screams as a means to identify tail biting events in weaner pigs. A large dataset of labeled videos for pig screams was created to find out when tail biting in four pens in one weaning compartment started and which pigs aggressively bit others. Intensive biters were identified by means of videos up to nine days before their identification by the farm staff during visual inspections. Thus, by using this method an earlier separation of pigs showing special interest in tail biting was possible compared to visual identification during daily inspections or the monitoring of the number of hanging tails. Therefore, this method represents a promising approach as a basis for an early warning system for tail biting.

Pig calls are often classified into low frequency and high frequency calls in specific situations, with the high-frequency calls (squeals and screams) indicating a more negative valence of the pigs, whereas low-frequency calls are mostly triggered in a neutral or more pleasant context^40,55^. The detection and use of pig screams (high-frequency calls) for different occasions were investigated in previous studies to identify pain-related or stressful situations, which have an impact on animal welfare^39,44–47,57,58^. Furthermore, the high-frequency sound structure and the relatively simple detection of a pig scream were similarly described as in our study and its relatively simple detection in the spectrogram was also confirmed. Reasons for the screams differed between those studies. Stress vocalizations were mostly provoked by immobilization^46,56^, mixing pigs from different pens^47^, restricted access to feed^46,47^, the different methods used for castrating pigs^56,57^, or observed ear biting events^45^. Unlike the aforementioned reasons, in the present study, screams under practical conditions labeled in audio data that were later evaluated in video recordings to allocate them to different causes showed that most screams were caused by tail biting behavior. Although scream duration differed between contexts, we found a high variation across the different contexts in this study, which made it difficult to distinguish contexts based on call duration only. The importance of call duration for classifying and detecting screams was also shown in other studies^45,47,56,57^. However, these studies used a multi-parametric approach combining temporal parameters such as call duration with spectral parameters (e.g., frequency distribution, formant pattern). Unfortunately, due to the overamplification of the majority of screams in our recordings and technical limitations of the microphones of the video cameras, we were not able to include spectral parameters in our analysis. Thus, further studies focusing on the acoustic structure of tail-biting screams in comparison to other contexts will be needed. Nevertheless, our study showed that video recordings can be used to detect an abnormal increase of pig screams. Thereby, detecting not only an increase due to tail-biting but also to aggression can be useful for management decisions.

Seven biting pigs were identified and separated from four pens during two batches. Due to the continuous monitoring of pig screams for detecting tail biting events and the individual pig identification by using colored ear tags, it was possible to identify the biters on video recordings in the daytime when the light was switched on. In other studies, pigs were marked individually on the back using animal marker pens or spray to enable identification at night^13,14,59^, but this needs to be repeated every couple of days^60^. Therefore, a light-independent and reliable animal identification, which is not labor-intensive, is needed.

Taylor et al.^25^ explained three different motivations of tail biting behavior, which may also explain the observed development of tail biting behavior. Regarding the pre-damage stage, where only gentle manipulation of another pig’s tail is described without visible damage to the tail, it is also expected that no painful scream caused by tail biting will be recorded. The damaging stage starts, probably during the non-aggressive tail-in-mouth behavior, as soon as the skin of the tail breaks and a tail lesion occurs, causing a higher interest in the tail from other pigs, especially when the wound is bleeding^25,27^. In the present study the first scream-detected tail biting events were performed by other pigs than the later separated biters. These removed biters had much higher interest in blood and can be assigned to the category of obsessive tail biters. However, the long observation period in this study (up to 10 days) underlines the difficulty for the farm staff to identify these biters during their daily animal inspections to separate them from the group as an intervention strategy.

Different approaches for early detection or the identification of early indicators for tail biting were taken, for example, observation of tail posture or activity of pigs^12,13,17–19^. Most studies were based on video analysis using only specific time windows for the evaluation. In the present study, audio files were analyzed continuously and reasons for screams were evaluated in the video according to an ethogram from the day a tail biter was removed from the pen retrospectively until no screams due to tail biting were observed for any one day. The sum of the tail lesion score and the percentage of hanging tails in a pen increased simultaneously up to six days before the separation of the biters. This is in accordance with Lahrmann et al.^17^ who investigated that more pigs had a hanging tail posture in pens with tail biting compared to control pens three days before a tail biting outbreak, which was defined as four pigs with a bleeding tail wound. In their study, one day before the outbreak, 33% of the pigs in a pen adopted a hanging tail posture. Similar results were also found by Zonderland et al.^20^ who found an increased chance of tail damage occurring two to three days after a hanging or tucked tail between the legs was observed. No identification of individual biters is possible by analyzing only tail posture of pigs at pen level, although the identification and removal of the biter was evaluated as an effective intervention strategy for reducing tail biting behavior^11,36^. Nonetheless, by continuously detecting pig screams, our study showed first screams due to tail biting up to 10 days prior to the removal of the biters. The sum of tail lesion scores and the percentage of hanging tails increased after the first screams caused by tail biting had been detected. Many other studies used only specific days and times (e.g., activity times) before a tail biting outbreak for behavioral observation^14,15,17,19^. According to Wilder et al.^61^, this leads to overestimation of the observed behaviors, whereas longer analyzed periods of time or even continuous sampling are more accurate, especially regarding irregular behaviors like tail biting. This was also demonstrated in our study for scream-detected tail biting events in the nighttime, for example, although nighttime is meant to be the resting time for pigs. On the other hand, in our study only tail biting events with a defense reaction in form of a scream due to painful biting were detected. Thus, events without a vocal response from the bitten pig were not evaluated and the number of silent biting events remains unclear.

A special challenge is localizing the scream and assigning it to one particular pen. Due to the open-plan structure of compartments in pig barns, screams from one pen are clearly audible in the neighboring pens. In our study, about a quarter of the detected screams originated from other pens although the sound was identical. For this purpose, Silva et al.^43^ developed an algorithm for localizing coughs to detect respiratory diseases in pig houses. By calculating the time delay between the different microphones in the compartment, it was possible to determine the pen the cough originated from. Another approach might be the comparison of loudness or signal-to-noise ratio, as it is expected to be highest in the pen where the scream occurred compared to the neighboring pens due to the physical distance^44^. When the pig scream detection is automated in the following step, such an algorithm for localizing the screams would be valuable, as it can indicate the pen where the biting occurred. Otherwise, the exact localization of the scream-detected tail biting behavior is missing. Additionally, in the last years acoustic cameras became more affordable. Acoustic cameras are able to visualize the sound location within the video recordings^62,63^. Thus, it allowed the monitoring of the behavior and sound location at the same time in the same video file for several pens.

Using audio data for automatically monitoring stress vocalization in pigs has already gained promising results regarding the identifying of stressful situations^46,47^. The simple and clear structure of the sounds makes them relatively easy to detect. Schön et al.^46^ developed a system called STREMODO (STREss MOnitor and DOcumentation unit), which combines Linear Prediction Coding (LPC) with an artificial neural network for detecting pig screams by microphone-generated audio files. Pig screams could be simply differentiated from non-stress vocalization or noise in the pig unit, which is in accordance with the present study. Nevertheless, no approach for detecting tail biting events by connecting pig screams to tail bites was made in that study. Vandermeulen et al.^47^ described stress vocalizations in more detail and additionally investigated the sound features that define a pig scream. Temporal and spectral parameters were used for training and validating an algorithm for automatic detection of pig screams. Due to the use of microphones without cameras in the two aforementioned studies, no assignment to the cause of the screams was possible and no relation to the occurrence of tail biting was made. However, those two studies confirm that pig screams can be clearly identified in sound data and that an automatic monitoring of stress vocalization is possible, thus providing a basis for identifying scream-detected tail biting events. The present study showed that the additional use of video cameras for evaluating the origin of screams and identifying tail biters would be a very useful tool for automatically detecting tail biting behavior and removing obsessive biters earlier. This could reduce the occurrence of tail damage and increase animal health and welfare. Continuous monitoring would be most valuable for farmers, as it analyzes pig behavior and vocalization in the nighttime and in between daily check-ups by the farmer.

## Conclusion

Pig screams could be clearly detected in audio files where most screams were triggered by ongoing tail biting events. As animals were marked individually, biter and victim pigs could be identified, which is valuable information for removing obsessive biters from the pen as an early intervention strategy to reduce further tail biting behavior and tail lesions. By using video recordings, intensive biters could be detected up to 9 days earlier than by visual inspection of the farm staff. The number of pigs with a hanging tail posture and tail lesions increased simultaneously after a higher number of pig screams were detected. Further investigations are needed to devise a detection algorithm for the automatic evaluation of animal vocalizations specifically focusing on detecting pig screams. The aim of future research is to record early warning signals and extract evidence-based video samples. These samples will then be used for straightforward identification of biters.

### Supplementary Information

Supplementary Information.

## Data Availability

The data presented in this study are available on request from the corresponding author. The data are not publicly available due to privacy concerns.
