# Supplementary material for: Detecting tail biters by monitoring pig screams in weaning pigs
Source: Sci Rep. 2024 Feb 24;14:4523. doi: 10.1038/s41598-024-55336-7 (PMC10894255; doi:10.1038/s41598-024-55336-7)
Supplement: Supplementary file 1 — Supplementary Information. [file 41598_2024_55336_MOESM1_ESM.pdf]

Detecting tail biters by monitoring pig screams in weaning pigs

Philipp Heseker[1,2,*], Tjard Bergmann[3], Marina Scheumann[3], Imke Traulsen[2,4], Nicole Kemper[1], and Jeanette Probst[1]

[1]Institute for Animal Hygiene, Animal Welfare and Farm Animal Behavior (ITTN), University of Veterinary Medicine Hannover, Foundation, Germany

[2]Department of Animal Sciences, Livestock Systems, Georg-August-University Goettingen, Germany

[3]Institute for Zoology, University of Veterinary Medicine Hannover, Foundation, Germany

[4]Institute of Animal Breeding and Husbandry, Christian-Albrechts-University Kiel, Germany

*Corresponding author: philipp.heseker@tiho-hannover.de

Supplementary information

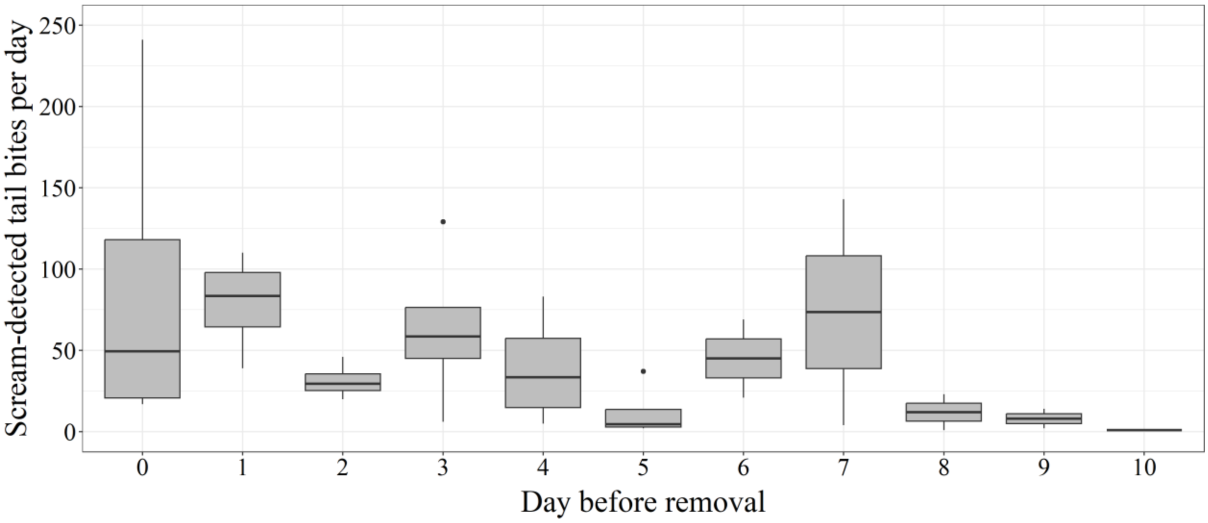

Supplementary Figure S1: Development of tail biting events per day detected by screams in the observation period in the four pens (pen 1 and 3: five days; pen 2: nine days; pen 4: ten days).
